# Production, Exacerbating Effect, and EV-Mediated Transcription of Hepatic CCN2 in NASH: Implications for Diagnosis and Therapy of NASH Fibrosis

**DOI:** 10.3390/ijms241612823

**Published:** 2023-08-15

**Authors:** Xinlei Li, Ruju Chen, Sherri Kemper, David R. Brigstock

**Affiliations:** 1 Center for Clinical and Translational Research, The Research Institute, Nationwide Children’s Hospital, Columbus, OH 43205, USA; rujuc9889@gmail.com (R.C.); sherri.kemper@nationwidechildrens.org (S.K.); david.brigstock@nationwidechildrens.org (D.R.B.); 2 Department of Surgery, Wexner Medical Center, The Ohio State University, Columbus, OH 43212, USA

**Keywords:** NASH, NAFLD, CCN2, CTGF, liver, fibrosis, fibrogenesis, collagen, extracellular vesicle, EV

## Abstract

Non-alcoholic steatohepatitis (NASH) is characterized by steatosis, hepatocyte ballooning, and inflammation and may progress to include increasingly severe fibrosis, which portends more serious disease and is predictive of patient mortality. Diagnostic and therapeutic options for NASH fibrosis are limited, and the underlying fibrogenic pathways are under-explored. Cell communication network factor 2 (CCN2) is a well-characterized pro-fibrotic molecule, but its production in and contribution to NASH fibrosis requires further study. Hepatic CCN2 expression was significantly induced in NASH patients with F3–F4 fibrosis and was positively correlated with hepatic Col1A1, Col1A2, Col3A1, or αSMA expression. When wild-type (WT) or transgenic (TG) Swiss mice expressing enhanced green fluorescent protein (EGFP) under the control of the CCN2 promoter were fed up to 7 weeks with control or choline-deficient, amino-acid-defined diet with high (60%) fat (CDAA-HF), the resulting NASH-like hepatic pathology included a profound increase in CCN2 or EGFP immunoreactivity in activated hepatic stellate cells (HSC) and in fibroblasts and smooth muscle cells of the vasculature, with little or no induction of CCN2 in other liver cell types. In the context of CDAA-HF diet-induced NASH, Balb/c TG mice expressing human CCN2 under the control of the albumin promoter exhibited exacerbated deposition of interstitial hepatic collagen and activated HSC compared to WT mice. In vitro, palmitic acid-treated hepatocytes produced extracellular vesicles (EVs) that induced CCN2, Col1A1, and αSMA in HSC. Hepatic CCN2 may aid the assessment of NASH fibrosis severity and, together with pro-fibrogenic EVs, is a therapeutic target for reducing NASH fibrosis.

## 1. Introduction

Nonalcoholic fatty liver disease (NAFLD) describes a spectrum of liver diseases ranging from simple steatosis caused by triglyceride accumulation in hepatocytes to nonalcoholic steatohepatitis (NASH), which features steatosis, hepatocyte ballooning, and inflammation, with or without fibrosis [1,2,3]. NAFLD/NASH affect millions of people globally and are an enormous healthcare burden: the global prevalence of NAFLD is estimated at 25% while that of NASH is 3–6% [4]. Patients with NASH may have no clinical symptoms, but over time, they can develop severe life-threatening conditions such as cirrhosis, hepatocellular carcinoma, and end-stage disease. It is expected that NASH-related chronic liver disease will soon become the leading cause of liver transplants in the United States [5,6]. The likelihood of developing NASH is increased in patients with obesity, type 2 diabetes, or metabolic syndrome [4]. However, approved drug therapies are lacking, and the primary treatment goal for all patients with NAFLD/NASH is weight loss through dietary modifications and exercise, although non-compliance with the required lifestyle changes seriously impedes the rates of NASH reversal [7,8,9,10].

Approximately 20% of patients with NAFLD develop NASH, and 20% of patients with NASH develop cirrhosis [11]. Liver fibrosis was reported to advance at a rate of 1 F stage per decade in 40% of NASH patients [4]. In light of several studies demonstrating that the severity of liver fibrosis in NASH is the best predictor of disease mortality [12,13,14], NASH fibrosis has become a central clinical and research focus [15,16,17]. The initiation and progression of NASH fibrosis is the result of activation and perpetuation of a highly matrigenic and fibrogenic phenotype in hepatic stellate cells (HSC), the principal fibrosis-producing cells in the liver that reside in the perisinusoidal space and account for about 5% of all cells in the liver [18]. HSC activation occurs in the setting of hepatocyte injury and inflammation that are hallmarks of NASH: multiple chronic hits to the liver (insulin resistance, obesity, diabetes, gut endotoxin, hyperlipidemia) cause lipotoxicity-induced oxidative or endoplasmic reticulum stress in hepatocytes while inflammatory infiltration of macrophages and neutrophils is driven by damage-associated molecular patterns from dying hepatocytes and inflammatory cytokines from hepatocytes or Kupffer cells (KC) [3,15,19]. The molecular and cellular changes associated with these chronic pathogenic pathways result in transcriptional alterations in quiescent HSC, resulting in their activation and unrelenting production and deposition of fibrous collagen in the interstitial space [20].

A key fibrogenic molecule that has become strongly implicated in driving fibrosis in multiple organs is cell communication network factor 2 (CCN2) [21,22], previously known as connective tissue growth factor (CTGF) [23]. In chronic human liver disease and rodent experimental fibrosis models, CCN2 is often produced by activated HSC in which it promotes activation, proliferation, chemotaxis, adhesion, integrin expression, fibrogenesis, and matrigenesis [24,25,26,27,28,29,30,31,32], although hepatocytes, cholangiocytes, vascular smooth muscle cells, and portal fibroblasts may produce CCN2 as well [21,32,33,34,35]. The biological importance of CCN2 is shown by hepatocyte overexpression of CCN2 in transgenic mice, which caused exacerbation of the fibrotic response to chemical toxins [36] and by the use of siRNA or hammerhead ribozyme to down-regulate CCN2 expression which reduced HSC activation and liver fibrosis in rodent models and highlighted CCN2 as a potential therapeutic target [37,38,39,40,41]. Whereas CCN2 has been strongly implicated in alcohol-, viral-, or toxin-mediated hepatic fibrosis in human or animal models, data are much less clear-cut in the context of NASH fibrosis. Since it has been emphasized that NASH fibrosis requires an improved understanding [3], this study was undertaken to clarify the localization and action of CCN2 in NASH fibrosis.

## 2. Results

### 2.1. CCN2 Is Induced in NAFLD Liver

**Figure 1:**
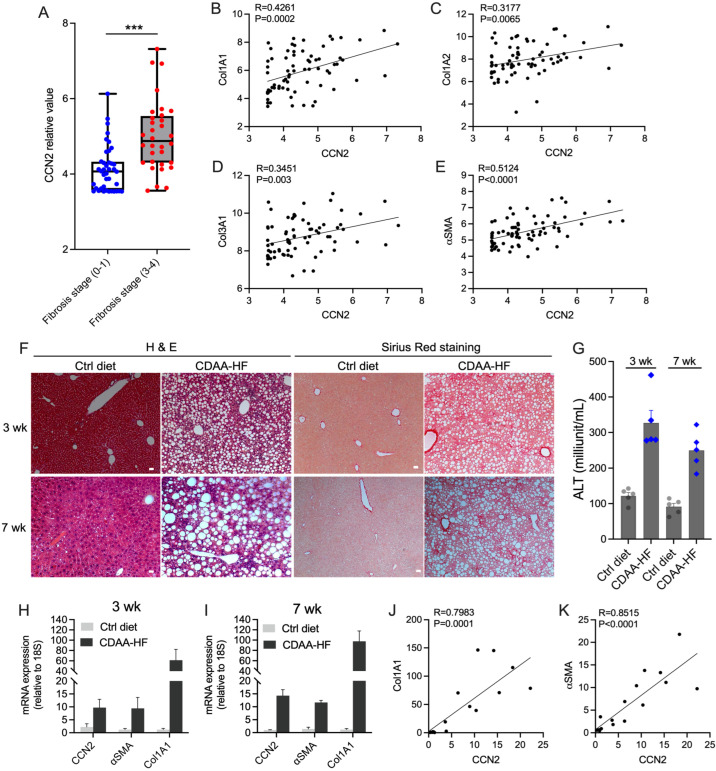
CCN2 is induced in NAFLD patients or diet-induced NASH in mice. Data from two groups of NAFLD patient cohort data (GSE49541) from mild NAFLD patients (fibrosis stage 0–1; *n* = 40) and advanced NAFLD patients (fibrosis stage 3–4; *n* = 32) were analyzed for (**A**) hepatic CCN2 expression as a function of liver fibrosis stage (blue, F0–F1; red, F3–F4) and for correlation between CCN2 expression and that of (**B**) Col1A1, (**C**) Col1A2, (**D**) Col3A1 or (**E**) αSMA. Swiss Webster mice were fed CDAA-HF diet for 3 or 7 weeks, after which they were sacrificed for collection of blood and liver tissue. Specimens were examined for (**F**) H&E staining for hepatic histology or Sirius red staining for collagen deposition, (**G**) serum ALT levels for liver injury, or (**H**,**I**) hepatic expression of CCN2, αSMA, or COL1A1 as assessed by RT-qPCR. Correlation between expression of CCN2 and (**J**) Col1A1 or (**K**) αSMA after 7 weeks of CDAA-HF feeding in Swiss Webster mice. Scale bar = 100 µm. *** *p* < 0.005.

Microarray results from two cohorts of NAFLD patients (GSE49541), including 40 patients with mild fibrosis and 32 with advanced fibrosis, as previously described [42,43], were retrieved from the GEO. As shown in Figure 1A, hepatic CCN2 expression was significantly induced in NAFLD patients with F3–F4 fibrosis compared to those with F0–F1 fibrosis. CCN2 expression was positively correlated with several fibrosis-related genes, including Col1A1, Col1A2, Col3A1, and αSMA (Figure 1B–E).

To further assess the expression of CCN2 in NAFLD pathogenesis, Swiss Webster WT mice were fed a control or CDAA-HF diet for up to 7 weeks for analysis of hepatic fibrosis-related molecules. Livers of WT mice fed the control diet exhibited a normal liver cellular architecture, whereas those fed the CDAA-HF diet showed lipid droplet accumulation in hepatocytes and interstitial collagen deposition as early as 3 weeks, which became more pronounced by 7 weeks (Figure 1F), consistent with the original description of this model [44]. Serum ALT was present at normal low levels in mice fed the control diet but was significantly elevated two–three-fold at 3 or 7 weeks in CDAA-HF-fed mice. After 3 or 7 weeks of CDAA-HF feeding, there was, respectively, a 7- or 14-fold increase in CCN2 expression, a 61- or 97-fold increase in Col1A1 expression, and a 10- or 11-fold increase in αSMA expression (Figure 1H,I). In CDAA-HF-fed mice, there was a strong positive correlation and high statistical significance between the increased level of CCN2 expression and that of Col1A1 or αSMA (Figure 1J,K).

### 2.2. CCN2 Localization in Normal or NASH Liver

**Figure 2:**
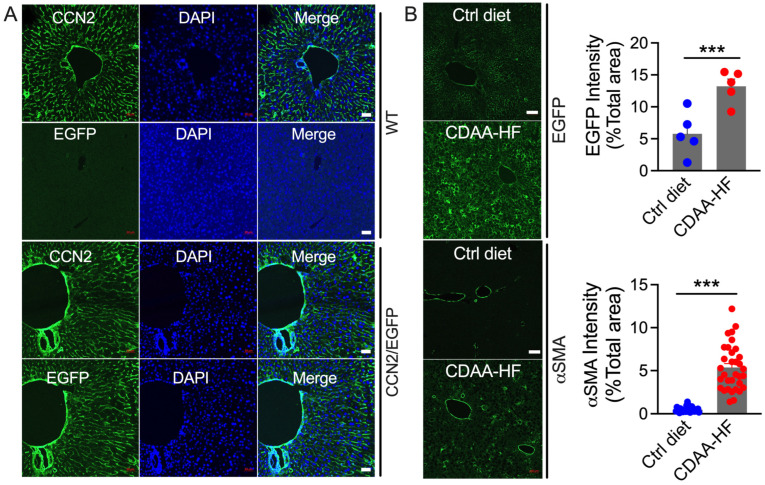
CCN2 localization in WT or CCN2/EGFP mice receiving control or CDAA-HF diet. (**A**) Swiss Webster WT mice (**top half**) or CCN2/EGFP reporter mice (**bottom half**) were maintained on control diet for 3 weeks prior to sacrifice and processing of livers for IHC for CCN2 or EGFP on sequential sections. Scale bar = 50 μm. (**B**) CCN2/EGFP reporter mice were fed control or CDAA-HF diet for 3 weeks, after which the mice were sacrificed, and livers sectioned for IHC of EGFP (left panels, top half) or αSMA (left panels, bottom half). Scale bar = 100 μm. Five fields from 3 mice in the control (blue) or CDAA-HF (red) groups were used for EGFP intensity quantification (right panel, top), and at least 30 fields from 5 mice in each group were used for αSMA quantification (right panel, bottom). *** *p* < 0.005.

CCN2 was detected in non-parenchymal regions, including sinusoidal areas and blood vessel walls, as assessed by CCN2 immunohistochemistry of 11-week Swiss Webster WT or CCN2-EGFP TG mice that had been fed a normal diet (Figure 2A). Moreover, the same staining pattern in CCN2-EGFP TG mice was seen using anti-EGFP, confirming that EGFP was a reliable surrogate marker for CCN2. As expected, no EGFP staining was seen in WT mice (Figure 2A). When WT or CCN2-EGFP TG mice were placed on a CDAA-HF diet for up to 7 weeks, there were no significant differences in body weight, liver weight, or expression of fibrotic genes, lipid metabolism genes, and proinflammatory genes between the two mouse strains (Supplementary Figure S1), further confirming that CCN2-EGFP TG mice were comparable to WT mice. When CCN2-EGFP TG mice were placed on a CDAA-HF diet for 3 weeks (by which time fibrosis is beginning to be initiated [44]), staining for hepatic CCN2 (EGFP) and αSMA significantly increased (Figure 2B), consistent with their WT counterparts (see Figure 1H–K).

**Figure 3:**
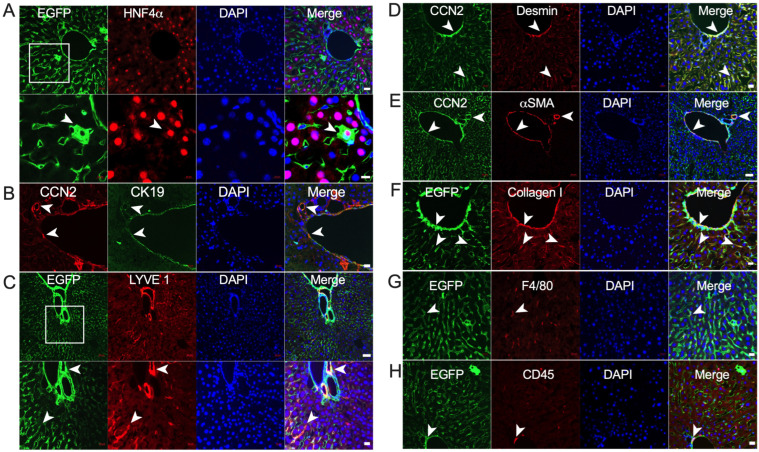
CCN2 localization in normal mouse liver. CCN2/EGFP reporter mice were fed control diet for 3 weeks, after which the mice were sacrificed and liver sections subjected to dual IHC staining as follows: (**A**) EGFP and HNF4α; (**B**) CCN2 and CK19; (**C**) EGFP and LYVE1; (**D**) CCN2 and desmin; (**E**) CCN2 and αSMA; (**F**) EGFP and collagen I; (**G**) EGFP and F4/80; and (**H**) EGFP and CD45. White arrowheads show representative colocalization of each pair of signals to the same cell. Scale bars: 20 µm, except for 10 µm for lower panel of (**A**) and 50 µm for upper panel of (**C**).

Although hepatic CCN2 levels were low in animals on a normal diet, we explored which hepatic cell types were responsible for its production under basal conditions. Using CCN2-EGFP TG mice, our approach was to co-stain for EGFP and a variety of cell-specific markers. With respect to epithelial cells, EGP was co-localized to no or very few HNF4α- or CES1- positive hepatocytes (Figure 3A and Supplementary Figure S2) or CK19-positive cholangiocytes (Figure 3B). For endothelial cells, there was pronounced EGFP co-localization within LYVE-1-positive vessels in the portal region (Figure 3C) and to luminal sinusoidal endothelial cells (LSEC) (Figure 3C). EGFP was also strongly localized in presumptive smooth muscle cells in periportal and pericentral vessel walls that stained for desmin, αSMA, and collagen, as well as in presumptive quiescent HSC in the sinusoidal areas that stained for desmin and collagen 1 but not for αSMA (Figure 3D–F). Finally, F4/80-positive macrophages showed very occasional and weak CCN2-staining in the sinusoidal regions (Figure 3G), while some CCN2 was also localized to some CD45-positive lymphocytes in vessel walls and sinusoids (Figure 3H), although each cell type occurred at very low frequency. Overall, these findings showed that in non-injured mice, hepatic CCN2 is produced at very low levels and is present primarily in the fibroblastic and endothelial populations with only very minor contributions from epithelial cells, macrophages, and lymphocytes.

**Figure 4:**
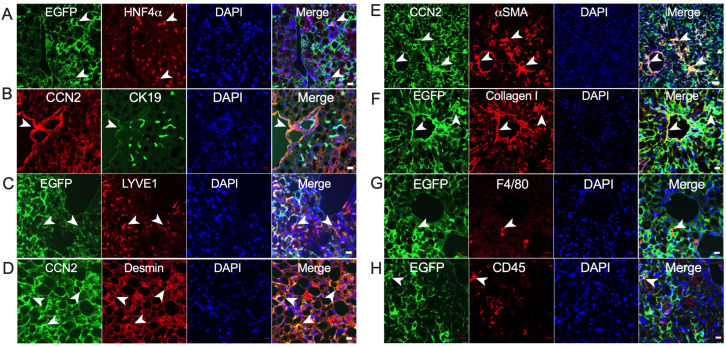
CCN2 localization in NASH mouse liver. CCN2/EGFP reporter mice were fed CDAA-HF diet for 3 weeks, after which the mice were sacrificed and liver sections subjected to dual IHC staining as follows: (**A**) EGFP and HNF4α; (**B**) CCN2 and CK19; (**C**) EGFP and LYVE1; (**D**) CCN2 and desmin; (**E**) CCN2 and αSMA; (**F**) EGFP and collagen I; (**G**) EGFP and F4/80; and (**H**) EGFP and CD45. White arrowheads show representative colocalization of each pair of signals to the same cell. Scale bars: 20 µm, except for 50 µm for (**E**).

We next assessed how CCN2 distribution was altered under NASH-like conditions using CCN2-EGFP TG mice that received a CDAA-HF diet for 3 weeks. Although the presence of hepatic steatosis caused major histological aberrations, there appeared to be only a modest increase in the frequency of EGFP or CCN2 signal in either HNF4α-positive fat-laden hepatocytes (Figure 4A) or periportal CK19-positive biliary epithelial cells (Figure 4B), although some CK19-positive cells were localized in the sinusoidal regions and were negative for EGFP. Strong EGFP staining occurred in LYVE1-positive endothelial cells in the sinusoids and vessels, but the pattern and frequency of staining were similar to that of control mice (Figure 4C). In contrast, there was a profound increase in overall staining for CCN2 or its EFGP surrogate in cells that had coincident staining for desmin, αSMA, and/or collagen (Figure 4D–F). These cells were identified as presumptive activated HSC in the sinusoidal region and as myofibroblasts in the vascular smooth muscle. The EGFP signal in F4/80-positive macrophages (Figure 4G) or CD45-positive lymphocytes (Figure 4H) remained low, although the latter population was somewhat increased in frequency compared to control mice.

### 2.3. CCN2 Transgene Exacerbates NASH Fibrosis

**Figure 5:**
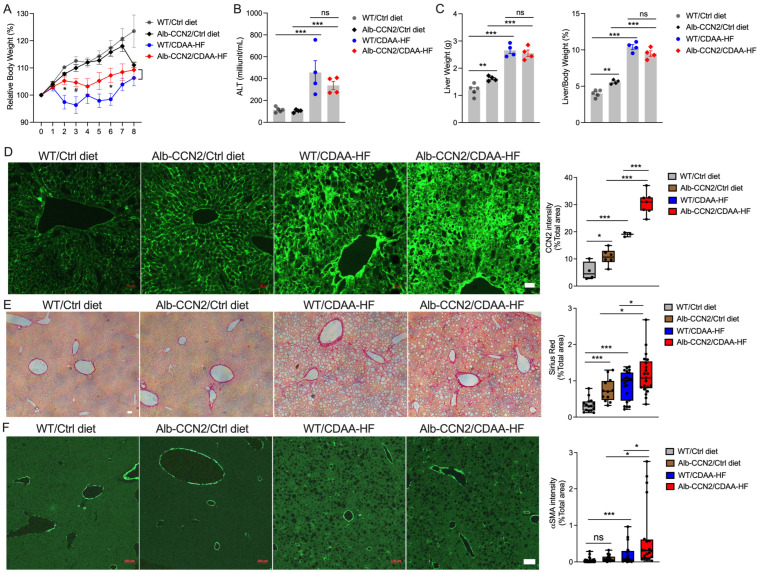
Alb-CCN2 TG mice have enhanced production of NASH-associated CCN2, αSMA, and fibrosis. WT or Alb-CCN2 TG Balb/c mice were placed on control diet or CDAA-HF diet, and (**A**) weekly body weights were measured over 8 weeks. Mice from WT group on control diet (grey circle),WT group on CDAA-HF diet (black diamond), TG group on control diet (blue circle), or TG group on CDAA-HF diet (red diamond) were then sacrificed for collection of blood and liver tissue, and specimens were examined for (**B**) serum ALT. (**C**) liver weight and liver index; (**D**) hepatic CCN2 by immunostaining; (**E**) collagen deposition by Sirius red staining; and (**F**) presumptive activated HSC and myofibroblasts by αSMA staining. Scale bar = 100 μm. * *p* < 0.05; ** *p* < 0.01; *** *p* < 0.005; ns, not significant.

We have previously shown that elevated hepatic CCN2 levels exacerbate experimental fibrosis in mice treated with chemical agents such as CCl_4_ or TAA [36]. To investigate potential interactions between hepatic CCN2 and NASH pathways, we compared liver pathology in WT versus Alb-CCN2 TG Balb/c mice maintained on a control or CDAA-HF diet. No significant differences in body weights from WT or Alb-CCN2 TG mice on the control diet were observed in the first 7 weeks, although Alb-CCN2 TG mice fed the control diet lost weight in Week 8 (Figure 5A). WT mice fed the CDAA-HF diet showed significant loss of body weight compared to the control-fed counterparts from Week 2 to Week 6. A similar but less severe weight loss occurred in CDAA-HF-fed Alb-CCN2 TG mice (Figure 5A). Serum ALT levels in either WT or Alb-CCN2 TG mice on the CDAA-HF diet were significantly elevated compared to their control diet counterparts, but there was no significant difference in ALT between Alb-CCN2 TG mice and WT mice that received the same diet (Figure 5B). Alb-CCN2 TG mice fed the control diet had significantly larger livers than WT mice, but there was no difference in liver mass of CDAA-HF-fed WT versus Alb-CCN2 TG mice, although CDAA-HF-fed mice from both groups had significantly larger livers than their control fed counterparts (Figure 5C, left panel). In view of the lower body weight in CDAA-HF-fed mice (Figure 5A), we also computed the liver index, which changed in the same manner as liver weights with the same significant differences between groups (Figure 5C, right panel). WT or Alb-CCN2 TG mice fed with the control diet exhibited a normal liver architecture, while those fed with the CDAA-HF diet showed apparent lipid droplet accumulation, but no visual differences between the two strains were observed (Supplementary Figure S3A). Although hyperglycemia occurs in some NASH models, blood glucose levels were significantly decreased in WT mice on the CDAA-HF diet for 8 weeks, but there was no change in Alb-CCN2 TG mice (Supplementary Figure S3B).

Expression of huCCN2 in the Alb-CCN2 TG mice was validated in our earlier study [36] and by RT-qPCR with human-specific CCN2 primers. Although mouse CCN2 transcript was significantly induced in either WT or Alb-CCN2 TG mouse liver upon CDAA-HF diet feeding, there was no significant difference in mouse CCN2 mRNA expression between the two strains on the same diets (Supplementary Figure S3C). However, the total CCN2 transcript level (assessed using primers that detect both native mouse CCN2 mRNA and transgenic human CCN2 mRNA) was dramatically elevated in either mouse strain, but the total CCN2 mRNA in Alb-CCN2 TG mouse liver was much higher than that in WT mouse liver on either control or CDAA-HF diet (Supplementary Figure S3C). Moreover, there was a significantly higher basal level of CCN2 immunostaining in Alb-CCN2 TG versus WT mice, and this difference was highly exaggerated when the mice were placed on a CDAA-HF diet, with considerable CCN2 staining in both parenchymal and non-parenchymal cells (Figure 5D). With respect to fibrosis-related readouts, Alb-CCN2 TG mice showed a higher basal level of collagen deposition (assessed by Sirius red staining) than WT mice on the control diet (Figure 5E). Staining for both collagen and αSMA was increased when each mouse strain was fed a CDAA-HF diet, and this was exacerbated in Alb-CCN2 TG versus WT mice (Figure 5E,F). Consistently, the Col1A1 transcript level in Alb-CCN2 TG mice on the CDAA-HF diet was much higher than that in WT mice on the same diet (Supplementary Figure S3C). The Alb-CCN2 TG model, thus, showed that HSC activation and fibrosis in NASH-like livers were causally linked to and enhanced by hepatic CCN2 protein levels.

### 2.4. CCN2 Is Induced in HSC In Vitro by EVs from Palmitic Acid-Treated Hepatocytes

**Figure 6:**
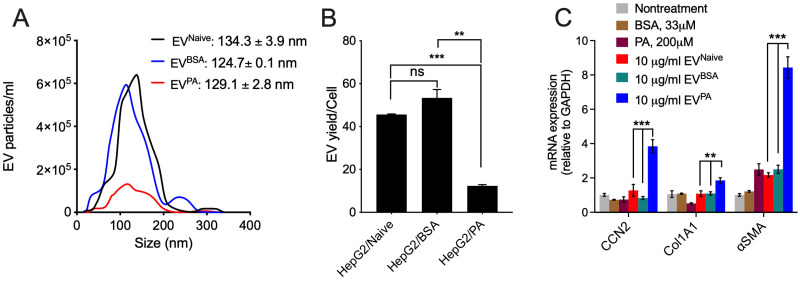
Induction of CCN2, Col1A1, or αSMA in human HSC by EVs from palmitic acid-treated human hepatocytes. HepG2 cells were cultured in serum-free medium containing BSA (33µM) or PA (200µM) for 48hr before collecting the supernatants for EV preparation by differential centrifugation. EV pellets were resuspended in DPBS and evaluated by NTA and BCA protein assay. (**A**) EV size distribution assessed by NTA. (**B**) EV yield expressed per HepG2 producer cell. (**C**) LX2 cells in serum-free DMEM were treated with 10 ug/mL of each EV type (EV^naive^, EV^BSA^, or EV^PA^) or 200 µM PA, or 33 µM BSA for 48hr prior to RT-qPCR detection of fibrosis-related gene expression. The figure shows the average of two independent experiments in which each RT-qPCR reaction was run in duplicate. ** *p* < 0.01; *** *p* < 0.005; ns, not significant.

As activated HSC appeared to be the predominant source of CCN2 in diet-induced NASH, we investigated the potential role of hepatocyte-derived extracellular vesicles (EVs) in driving CCN2 production in HSC because EVs have emerged as important mediators of intercellular communication during various pathogenic processes. Human HepG2 hepatocytes were treated with PA (or BSA carrier control) for 48 h, after which their released EVs were purified from the conditioned medium. As assessed by NTA, there was no significant difference between the diameters of EV^naive^ and EV^PA^ (134.3 ± 3.9 nm vs. 129.1 ± 2.8 nm, respectively), but EV^BSA^ were somewhat smaller (124.7 ± 0.1 nm) (Figure 6A). The yields per cell of EV^naive^ and EV^BSA^ were not significantly different, whereas the yield of EV^PA^ per cell was significantly reduced (Figure 6B), although sufficient numbers of EVs were nonetheless readily recovered for further analysis. Accordingly, EV^PA^ was found to stimulate the expression of CCN2, Col1A1, or αSMA in LX2 HSC by four-, two- or eight-fold, respectively (Figure 6C). This gene induction was significantly greater than that in response to EV^BSA^ or EV^naive^, which had no effect on CCN2 or Col1a1 expression and which caused only a three-fold increase in expression of αSMA mRNA, the latter of which was also seen in response to direct treatment of the cells with PA. Other than this, neither PA nor BSA caused changes in fibrosis-related gene expression in LX2 cells (Figure 6C). Hence hepatocytes loaded with free fatty acids produced a unique population of pro-fibrogenic EVs that drove expression of CCN2 and other fibrotic genes in HSC that may contribute to NASH fibrosis and likely mechanistically contributed to our observations of CCN2 production in the mouse models reported above.

## 3. Discussion

Liver fibrosis is an important clinical feature of NASH because it is highly predictive of the progression to mortality. Improved understanding of mechanisms of fibrosis development and progression in the setting of NASH are crucial for a more complete understanding of disease pathogenesis as well as providing new leads for therapeutic options. Since prior studies regarding CCN2 production in NASH have led to varying observations and conclusions, we employed validated transgenic systems for reporting CCN2 localization and function. Indeed, the robustness of our ability to accurately detect CCN2 was confirmed by the identical localization of fluorescent signals using CCN2 IHC compared to EGFP IHC. An added strength of our study was the use of fluorescent antibody probes and confocal microscopy, which permitted highly sensitive detection and localization of CCN2, even in control mice in which basal CCN2 levels in some cell types are extremely low. Importantly, IHC provided important information regarding CCN2 protein levels and localization and was a more dynamic approach than commonly used RNA quantification measurements which have limitations with respect to the origin and translational status of the encoded transcript, especially in the case of CCN2, which is a very complex matricellular molecule that may show discordant RNA and protein levels. While our CCN2 mRNA findings were in general agreement with our CCN2 protein findings, they were more limited with respect to cellular origin or localization. Our IHC approach showed that CCN2 in NASH livers was principally attributed to its increased presence in HSC and to fibroblasts and smooth muscle cells of the vasculature: these cells exhibited a substantially stronger CNN2 signal in mice fed CDAA-HF diet compared to the signal in their counterparts in mice that received the control diet. Endothelial cells also stained prominently for CCN2, but the signal appeared to be constitutive and generally unaltered by exposure to the CDAA-HF diet. Epithelial cells and lymphoid cells stained weakly and/or infrequently for CCN2 in control animals; these signals were somewhat more prevalent in CDAA-HF-fed mice, but the frequency of CCN2-positive cells remained low. CCN2-positive macrophages were very sparse in mice receiving either diet. Furthermore, the study of Alb-CCN2 mice allowed us to demonstrate that hepatic CCN2 production during NASH drives and exacerbates HSC activation and collagen deposition.

While TGF-β, IL-6, fatty acids, or angiotensin II produced in NASH may be involved in stimulating CCN2 production among the various hepatic cell types [45], our results show that the exposure of hepatocytes to free fatty acid results in their production of pro-fibrogenic EVs that induce transcription of CCN2 and other fibrosis-related molecules (Col1A1, αSMA) in HSC. Similar recent studies have also shown that such EV populations induce HSC activation and fibrogenic gene expression [46,47,48], but CCN2 mRNA was not previously identified as a transcriptional target. Our data, thus, points to a novel mechanism by which CCN2 transcription is modulated in HSC during NASH fibrosis. Our future studies will aim to determine whether CCN2 is similarly EV-regulated in fibroblasts or vascular smooth muscle cells, to identify EV components responsible for CCN2 transcription, and to establish whether EV-related mechanisms are operative in our in vivo models. It is notable that inflammation and insulin resistance in NASH are also regulated by EVs from fatty acid-treated hepatocytes [49], highlighting the involvement of such EVs in multiple aspects of NASH pathogenesis. Targeting the production or action of these hepatocyte-derived pathogenic EVs may offer interesting new leads for NASH therapy.

CCN2 is strongly pro-fibrotic as it has profound actions that lead to or enhance HSC activation, including cell adhesion, proliferation, locomotion, and fibrogenesis [28]. With respect to CCN2 fibrotic activity in NASH, future studies will need to address the precise CCN2 molecular configuration (monomer vs. dimer), the role played by individual modules in the CCN2 protein, the role of CCN2 proteolytic processing to generate low-mass bioactive isoforms and identification of CCN2 cell surface receptors and signaling pathways. However, our prior studies have shown that activated HSC in vitro engages full-length CCN2 or proteolytic fragments of CCN2 via integrins, heparin sulfate proteoglycans, and low-density lipoprotein-related protein [31,50] and similar mechanisms are likely in NASH in vivo. Our data showing that activated HSC is a principal source of CCN2 are consistent with prior but very limited studies regarding the localization of CCN2 in the extracellular matrix (ECM). For example, in human NASH livers, CCN2 localized to areas of ECM in the portal tracts and fibrous septa, where it was predominantly associated with sites of fibrogenesis (i.e., containing activated HSC), resulting in a positive correlation between CCN2 and fibrosis stage [51]. C57BL6/J mice fed a methionine and choline-deficient (MCD) diet for 8 weeks showed typical NASH-like morphology with increased CCN2 immunostaining in the fibrous septa [52]. In vitro, increased CCN2 mRNA expression or protein production was shown to result from the exposure of cultured Day 4 primary rat HSC to glucose or insulin to mimic, respectively, the hyperglycemia or insulin resistance that are frequently seen in NASH patients [51]. Since our in vivo model did not cause hyperglycemia, this confounding variable will be included in future studies.

Although our data reveal minimal CCN2 production by hepatocytes in NASH fibrosis, other studies have implicated hepatocytes instead of HSC, but this often happens during earlier non-fibrotic stages of the disease and/or in concert with other injurious pathways. For example, CCN2 mRNA expression was greater in inflamed steatotic livers of non-fibrotic human NASH compared to its low levels in non-steatotic or steatotic livers [53]. Since treatment of primary human hepatocytes with palmitic or oleic acids did not alter CCN2 production, it was speculated that elevated expression of CCN2 and TGF-β during the inflammatory phase of NASH renders the liver more susceptible to developing fibrosis. This type of interaction may have also contributed to increased NASH fibrosis in our Alb-CCN2 TG mouse model, although other explanations are possible. In primary human hepatocytes, TGF-β-dependent and -independent induction of CCN2 mRNA or protein was blocked by adiponectin [53,54], an effect attributed to, first, the induction by adiponectin of BMP and activin membrane-bound inhibitor (BAMBI), a TGF-β pseudoreceptor [54] and, second, pathways involving peroxisome proliferator-activated receptor α (PPARα), a downstream effector of adiponectin receptor 2 (AdipoR2) [53]. The relevance of these findings to NASH is that systemic adiponectin levels progressively diminish in human steatosis and NASH [55,56,57,58], while AdipoR2 exhibits suppressed expression in human NASH livers and in human hepatocytes from NASH patients [53,59,60,61]: a collectively diminished adiponectin axis in the liver may account for increased CCN2 (and TGF-β) in non-fibrotic NASH.

In the fa/fa Zucker rat model of obesity and diabetes caused by leptin receptor deficit, CCN2 mRNA and protein were enhanced by 35 weeks of age in fa/fa rats but not in lean fa/fa littermates [51]. However, liver histology and expression of collagen or TGF-β1 in fa/fa rats were normal and comparable to Fa/fa rats leading the investigators to conclude that CCN2-mediated fibrosis required the presence of other pathological hits such as steatosis or inflammation [51]. Indeed this may well account for the exacerbating effect of CCN2 on fibrosis in Alb-CCN2 TG mice because this and several other CCN2 transgenic systems have been shown to be relatively inconsequential unless other interacting pathways (injury, development) are concurrently present [62]. In rhesus monkeys fed HFD for 2 years, expression of hepatic CCN2, TGF-β αSMA, and Smad 3 were increased in simple steatosis compared to healthy controls and in fibrosing NASH compared to simple steatosis, with CCN2 correlating positively with the severity of fibrosis [63]. Activation of the hepatic TGF-β axis in this system was attributed to Yes-associated protein (YAP), which was induced as a function of injury severity in the nuclei of hepatocytes, perivascular cells, and bile duct cells [63]. A 2.7-fold increase in CCN2 expression was documented by microarray analysis of hepatocytes isolated from 35-week Pten knock-out mice, which develop NASH-like liver pathology leading the authors to speculate that CCN2 was involved in NASH fibrosis or cancer [64].

In a C57BL6/J mouse model of NASH resulting from a 20-week high-fat diet (HFD) and streptozotocin-induced diabetes mellitus (DM) over the last 5 weeks, hepatic CCN2 mRNA concentrations did not show statistically significant differences compared to chow-fed animals, but nonetheless, they trended upward and were correlated with increased collagen and TIMP-1 expression [65]. HFD alone or BM alone did not elicit any significant changes in hepatic CCN2 mRNA or protein, but upward trends were still noted in each group [65]. In contrast, HFD + DM mice demonstrated a significant increase in hepatic CCN2 protein, which was localized both to hepatocytes and to the central veins and portal tracts within the fibrotic regions [65]. Alms1 mutant (foz/foz) C57BL6/J mice fed a high-fat diet for 24 weeks led to diet-induced NASH that included hepatocyte ballooning, inflammation, high NAFLD activity, and ALT levels, as well as severe hepatic fibrosis and increased hepatic expression of PDGF-α, αSMA, collagen-1α, and CCN2, the latter of which was associated with CD-147 expression around hepatocytes and increased MMP activity and expression of MMP-2 and -9 [66]. These changes were highly attenuated in foz/foz Balb/c mice and attributed to strain differences in diabetes and inflammatory phenotype [66].

Several studies have demonstrated attenuated CCN2 expression as part of the response to therapeutic agents when tested in rodent models of NASH. For example, rosiglitazone decreased inflammation in an MCD diet-induced model of steatohepatitis in rats [67] and steatosis and serum transaminases in the one-year follow-up of the FLIRT trial of human NASH [68]. While there was no improvement of NASH fibrosis in the FLIRT study [68], hepatic fibrosis was, nonetheless, improved by rosiglitazone in MCD diet-fed C57BL6/J mice, and this was attributed to activation of PPARγ, which caused reduced HSC activation and suppressed expression of TGF-β1 and CCN2 [52]. The same mouse model was used to show that quercetin, a common flavonoid in human diets, suppressed NASH liver injury, inflammation, fibrosis, and fibrosis-related molecules, including CCN2 [69]. Administration of caffeine to rats that were fed a high fat, high sucrose, high cholesterol diet to induce NASH resulted in diminished fibrosis, collagen deposition, and expression of hepatic TGF-β, CCN2, and αSMA [70].

Finally, some studies have provided evidence in support of CCN2 as a biomarker or therapeutic target in NASH. For example, our analysis of human NASH gene datasets showed that hepatic CCN2 mRNA expression is positively correlated with the hepatic fibrosis stage, consistent with several earlier studies of CCN2 protein as assessed by IHC [51,63]. Moreover, in NAFLD patients, serum CCN2 was higher than in healthy controls, allowed discrimination between patients with no, mild or advanced fibrosis, and was an independent predictor of fibrosis stage [71]. After brain death, hepatic CCN2 was transiently increased in NASH livers but reduced in non-steatotic livers, leading to the proposal that hepatic CCN2 levels have clinical utility for helping evaluate the severity of steatosis in brain-dead donors whose livers are being considered for transplantation [72]. Finally, our data show that CCN2 production is central to the fibrotic response in NASH and that elevated hepatic CCN2 can drive more pronounced aspects of NASH fibrosis, highlighting its potential as a target by which NASH fibrosis could be suppressed and thus reducing the associated risk of increased disease severity and death. Indeed, in an HFD + DM model of NASH in C57BL6/J mice, administration of a CCN2 neutralizing resulted in reduced levels of fibrosis and expression of fibrosis-related proteins and signaling pathways [73]. Hepatic levels of mRNA for cell communication network factor 3 (CCN3), which is often expressed reciprocally to and is an antagonist of CCN2 [74,75], was suppressed in the NASH model, and this was reversed by CCN2 antibody administration [73].

In conclusion, these data extend our knowledge of hepatic CCN2 in NASH fibrosis by showing that CCN2 expression is associated with liver fibrosis severity, is principally induced in activated HSC and myofibroblasts, and exacerbates the degree of HSC activation and fibrosis in the context of NASH injury. Based on prior studies, the transcriptional activation and full fibrogenic actions of CCN2 likely require the presence of injury-related pathways such as steatosis, inflammation, diabetes, or other metabolic disorders. CCN2 is, thus, a rational diagnostic or prognostic indicator and therapeutic target for the fibrotic stages of NASH, which predispose to more advanced and life-threatening injury.

## 4. Materials and Methods

### 4.1. Non-Alcoholic Steatohepatitis (NASH) Disease Model

Animal protocols were approved by the Institutional Animal Care and Use Committee of Nationwide Children’s Hospital (Columbus, OH, USA). Male Swiss Webster wild type (WT) or transgenic (TG) mice expressing enhanced green fluorescent protein (EGFP) under the control of the promoter for cellular communication network factor 2 (CCN2)(CCN2-EGFP; [76]; 8 weeks old; *n* = 5 per group), or male Balb/c WT or TG mice expressing human CCN2 governed by the albumin promotor (Alb-CCN2; 8 weeks old; *n* = 5 per group) [36] were given either choline-deficient or amino-acid defined (CDAA) diets with high (60%) fat (CDAA-HF (Catalog #A06071302, Research Diets Inc., New Brunswick, NJ, USA) for up to 8 weeks to induce steatohepatitis with fibrosis as previously described [44] or normal (low fat) chow food (2020X, Inotiv, West Lafayette, IN, USA). The formula comparison of the two diets is shown in Supplemental Table S1. Body weights were recorded weekly until the conclusion of each feeding regime. Previous studies showed that in mice subjected to the CDAA-HF diet, increased serum ALT, hepatic collagen deposition, and expression of TGFβ1, Col1A1, and TIMP-1 mRNA were apparent by around Week 4, and these features persisted and were more robust by Week 8 [44]. Based on this information and our own pilot studies, liver tissues were harvested at Weeks 3, 7, and/or 8. To accomplish this, mice were anesthetized with 12.5 mg/mL ketamine and 1.5 mg/mL xylazine; blood was drawn by cardiac puncture, and the liver was perfused sequentially with PBS and 4% paraformaldehyde (Sigma-Aldrich, St. Louis, MO, USA) through the left heart ventricle. The perfused livers were collected and weighed after the mice were sacrificed. Liver lobes were then used for paraffin embedding or frozen tissue sectioning. In some cases, one lobe of the liver was tied off with suture material after PBS perfusion, removed immediately, and snap-frozen in liquid nitrogen for subsequent RNA extraction.

### 4.2. Histology

Perfused mouse livers were fixed and embedded in paraffin or subjected to frozen tissue embedding in an O.C.T compound. Paraffin sections of 5 μm thickness were cut and stained with H and E for histology or with 0.1% Sirius Red (Sigma-Aldrich) for detection of collagen. Paraffin or frozen sections of 5 μm thickness were subjected to immunofluorescent staining. Positive signals were quantified by ImageJ (NIH. Bethesda MD).

### 4.3. Immunofluorescence

Frozen or paraffin-embedded mouse liver sections were incubated with primary antibodies to F4/80 (Catalog #70076S,1:250, Cell Signaling), CD45 (Catalog #70257S, 1:500, CST), alpha smooth muscle actin (αSMA; catalog # A700-082-T, 1:500; Thermo Fisher Scientific, Waltham, MA, USA), EGFP (Catalog #632375, 1:10,000, mouse monoclonal, Takara Bio, Palo Alto, CA, USA), CCN2 (Catalog #MA5-31420, 1:400; mouse monoclonal, Thermo Fisher Scientific), desmin (eFluor™ 660 conjugated, catalog #50-9747-80, 1:25, Invitrogen, Waltham, MA, USA), lymphatic vessel endothelial hyaluronan receptor 1 (LYVE1; catalog #67538 1:250, CST), hepatocyte nuclear factor 4 alpha (HNF4α; catalog #A20865, 1:250, Abclonal, Woburn, MA, USA), carboxylesterase 1 (CES1; catalog #ab45957, 1:500, Abcam), cytokeratin 19 (CK19, catalog #TROMA-III, 1:200, Developmental Studies Hybridoma Bank, University of Iowa, Iowa City, IA, USA), or collagen 1a1 (Col1A1; catalog #ab34710, 1:250, Abcam), followed by Alexa Fluor 488 goat-anti rat IgG, Alexa Fluor 488 goat-anti mouse IgG, Alexa Fluor 568 goat-anti mouse IgG, Alexa Fluor 568 goat-anti rabbit IgG, or Alexa Fluor 488 goat-anti-rabbit IgG (all at 1:500; Thermo Fisher Scientific) for 1 h at room temperature. The slides were counterstained with 4′,6-diamidino-2-phenylindole (DAPI, 1:1000; Thermo Fisher Scientific) and mounted in glycerol gel. The slides were examined by confocal microscopy, and positive signals were quantified by ImageJ.

### 4.4. Liver Function and Glucose Tests

Blood was drawn at the end of this experiment and allowed to clot. Serum was obtained by centrifugation at 5000× *g* for 10 min and stored at −80 °C before use. Each serum sample was diluted 10-fold with PBS and assayed for alanine aminotransferase (ALT) using a commercial kit (Sigma-Aldrich) following the manufacturer’s instructions. Serum glucose levels were measured using the glucose oxidase–peroxidase method with a glucose assay kit (catalog #E-BC-K234-M, Elabscience, Houston, TX, USA).

### 4.5. Patient Cohort Sequencing Data Analysis

Two groups of NAFLD patient cohort data (Gene Expression Omnibus (GEO) accession number GSE49541) from mild NAFLD patients (fibrosis stage 0–1; *n* = 40) and advanced NAFLD patients (fibrosis stage 3–4; *n* = 32) as previously reported [42,43] were retrieved and analyzed with the online program GEO2R. Results were corrected by Benjamini–Hochberg’s method to control the false discovery rate at 5%.

### 4.6. Isolation and Characterization of Extracellular Vesicles (EVs) from Palmitic Acid-Treated Hepatocytes

A total of 1mM palmitic acid (PA) stock was prepared conjugated with lipid-free bovine serum albumin (BSA) (6:1 ratio of PA:BSA), as described [77]. Human hepatocyte HepG2 cells (HB-8065, American Type Culture Collection, Manassas, VA, USA) were cultured as we have previously described [78]. Cells were then switched for 48 h to serum-free medium alone or which contained 200 µM PA or 33 µM BSA. Using our established methodology [78], EVs were then purified from the conditioned medium by sequential differential centrifugation (300× *g* for 10 min, 2000× *g* for 20 min, 10,000× *g* for 30 min, and two steps of 100,000× *g* for 70 min), resuspended in Dulbecco’s phosphate-buffered saline (DPBS; Thermo Fisher Scientific), and characterized for size and frequency by the nanosight tracking analysis (NTA) and for protein concentration by Pierce bicinchoninic acid (BCA) assay (Thermo Fisher Scientific). Next, human LX2 HSC were cultured as described [79], placed in serum-free Dulbecco’s Modified Eagles Medium (DMEM; Thermo Fisher Scientific), and incubated for 48 h with 10 µg/mL EVs obtained from the three treatment groups (EV^naive^, EV^PA^, EV^BSA^), 200 µM PA, or 33 µM BSA. Cells were then processed for RNA expression analysis.

### 4.7. RNA Extraction and Real-Time Quantitative Polymerase Chain Reaction (RT-qPCR)

Total RNA from liver tissues or LX2 cells was extracted using a miRNeasy mini kit (Qiagen, Germantown, MD, USA) and subjected to RT-qPCR for transcript evaluation using an Eppendorf Mastercycler System or Bio-Rad CFX Connect Real-Time PCR System and one-step iTaq Universal SYBR Green Supermix (Bio-Rad, Hercules, CA, USA). Primers are shown in Table 1 and Supplemental Table S2. Each reaction was run in duplicate, and samples were normalized to 18S rRNA or GAPDH.

### 4.8. Statistical Analysis

Experiments were performed at least twice in duplicate or triplicate, with data expressed as mean ± SEM. Fluorescence images were scanned and quantified using ImageJ. Data from RT-qPCR and imaging were analyzed by Student’s *t*-test. The correlations between CCN2 and COL1A1, COL1A2, COL3A1, or αSMA were analyzed with Prism 9. Pearson correlation coefficients and *p*-values < 0.05 were considered statistically significant.

## Figures and Tables

**Table 1:** Primers used for qRT-PCR.

Gene ID	Accession No.	Primer		Length (bp)
		Fwd Seq (5′-3′)	Rev Seq (5′-3′)	
CCN2 (mouse)	NM_010217	CACTCTGCCAGTGGAGTTCA	AAGATGTCATTGTCCCCAGG	111
Col1A1 (mouse)	NM_007742	GCCCGAACCCCAAGGAAAAGAAGC	CTGGGAGGCCTCGGTGGACATTAG	148
GAPDH (human and mouse)		TGCACCACCAACTGCTTAGC	GGCATGGACTGTGGTCATGAG	87
Col3A1 (mouse)	NM_009930	GCCCACAGCCTTCTACACCT	GCCAGGGTCACCATTTCTC	110
αSMA (mouse)	NM_007392	GGCTCTGGGCTCTGTAAGG	CTCTTGCTCTGGGCTTCATC	148
CCN2 (human)	NM_001901	AAAAGTGCATCCGTACTCCCA	CCGTCGGTACATACTCCACAG	109
Col1A1 (human)	NM_000088	GAACGCGTGTCATCCCTTGT	GAACGAGGTAGTCTTTCAGCAACA	91
αSMA (human)	NM_001613	GTGTTGCCCCTGAAGAGCAT	GCTGGGACATTGAAAGTCTCA	109

## Data Availability

The data presented in this study are available on request from the corresponding author.

## Supplementary Materials

The following supporting information can be downloaded at: https://www.mdpi.com/article/10.3390/ijms241612823/s1.
