# Supplementary material for: Production, Exacerbating Effect, and EV-Mediated Transcription of Hepatic CCN2 in NASH: Implications for Diagnosis and Therapy of NASH Fibrosis
Source: Int J Mol Sci. 2023 Aug 15;24(16):12823. doi: 10.3390/ijms241612823 (PMC10454308; doi:10.3390/ijms241612823)
Supplement: Supplementary file 1 [file ijms-24-12823-s001.zip › ijms-2386519-supplementary.pdf]

Supplementary materials

Table S1. Formulation of control and CDAA-HF diets.

Nutrients		CDAA-HF	Ctrl diet
		A06071302	2020X
**Protein**	%	23.1	19.4
Carbohydrate	%	26.3	47
Fat	%	35.7	6.5
Fiber	%	6.6	2.7
Methionine	%	0.1	0.5
Choline	g/kg	0	1.2
Calories from Protein	%	18	24
Calories from Carbohydrate	%	20	60
Calories from Fat	%	62	16
Energy density	Kcal/g	5.2	3.1

Table S2. Additional primers used for RT-qPCR.

Gene ID	Accession No.	Primer		Length(bp)
		Fwd Seq (5'-3')	Rev Seq (5'-3')	
CCN2 (human and mouse)		AATGCTGCGAGGAGTGGGT	GGCTCTAATCATAGTTGGGTCT	115
mMCP-5/CCL12	NM_011331	GTCCTCAGGTATTGGCTGGA	GGGTCAGCACAGATCTCCTT	181
mMIP-1a/CCL3	NM_011337	TGAAACCAGCAGCCTTTGCTC	AGGCATTCAGTTCCAGGTCAGTG	125
mRANTES/CCL5	NM_013653	AGATCTCTGCAGCTGCCCTCA	GGAGCACTTGCTGCTGGTGTAG	170
mTIMP-1	NM_001044384	CCTATAGTGCTGGCTGTGGG	GCAAAGTGACGGCTCTGGTA	136
mSREBP1a	NM_011480	GGCCGAGATGTGCGAACT	TTGTTGATGAGCTGGAGCATGT	69
mSREBP1c	NM_001358314	GGAGCCATGGATTGCACATT	GGCCCGGGAAGTCACTGT	70
mFASN	NM_007988	GCTGCGGAAACTTCAGGAAAT	AGAGACGTGTCACTCCTGGACTT	84
mSCD1	NM_009127	TTCTTCTCTCACGTGGGTTG	CGGGCTTGTAGTACCTCCTC	130

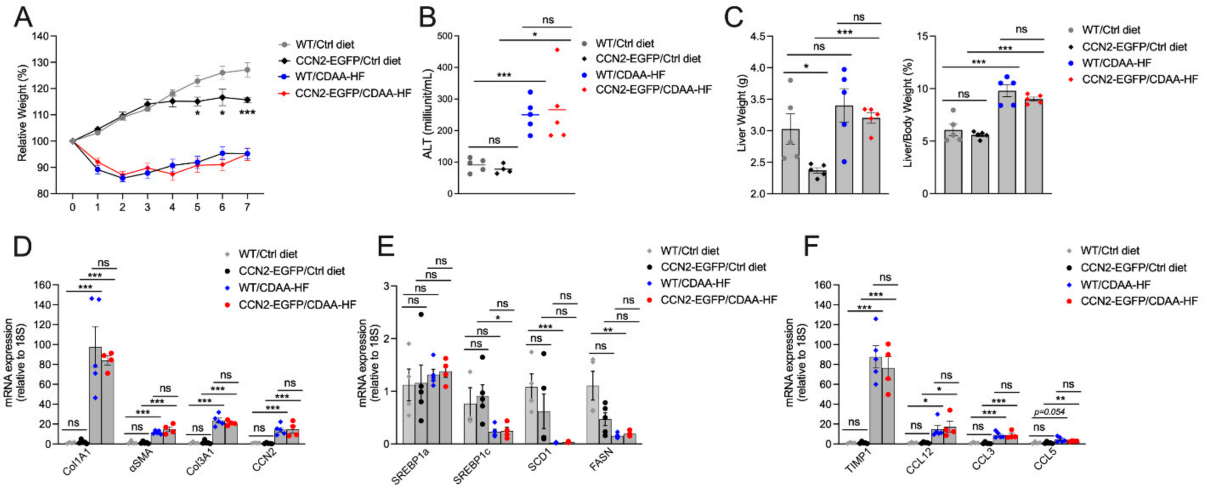

Figure S1. Swiss Webster WT and CCN2-EGFP TG mice exhibit comparable responses upon CDAA-HF diet feeding. 8-week-old Swiss Webster WT or CCN2-EGFP TG mice were fed with control or CDAA-HF diet for 7 weeks. (**A**) Body weight was recorded every week. (**B**) Serum ALT levels were measured at the end of 7 weeks. (**C**) Liver weights and liver index (liver/body weight) were measured after the mice were sacrificed. (**D**) Liver fibrotic genes, (**E**) lipid metabolism-related genes, and (**F**) proinflammatory cytokine genes were measured by RT-qPCR. ns, no significance, * P<0.05, ** P<0.01, *** P<0.005.

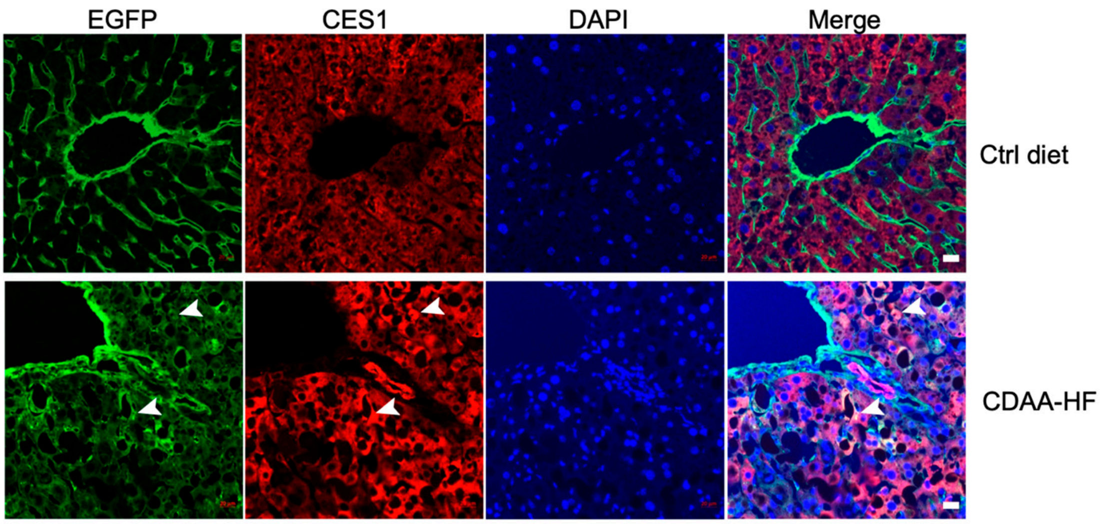

Suppl. Figure 2.

Figure S2. Hepatic CES1 localization. Sections of livers from CCN2/EGFP reporter mice fed control diet or CDAA-HF diet for 3 weeks were stained for EGFP and CES1. Arrowheads show areas of co-localization of the two markers. Scale bar=20 μm.

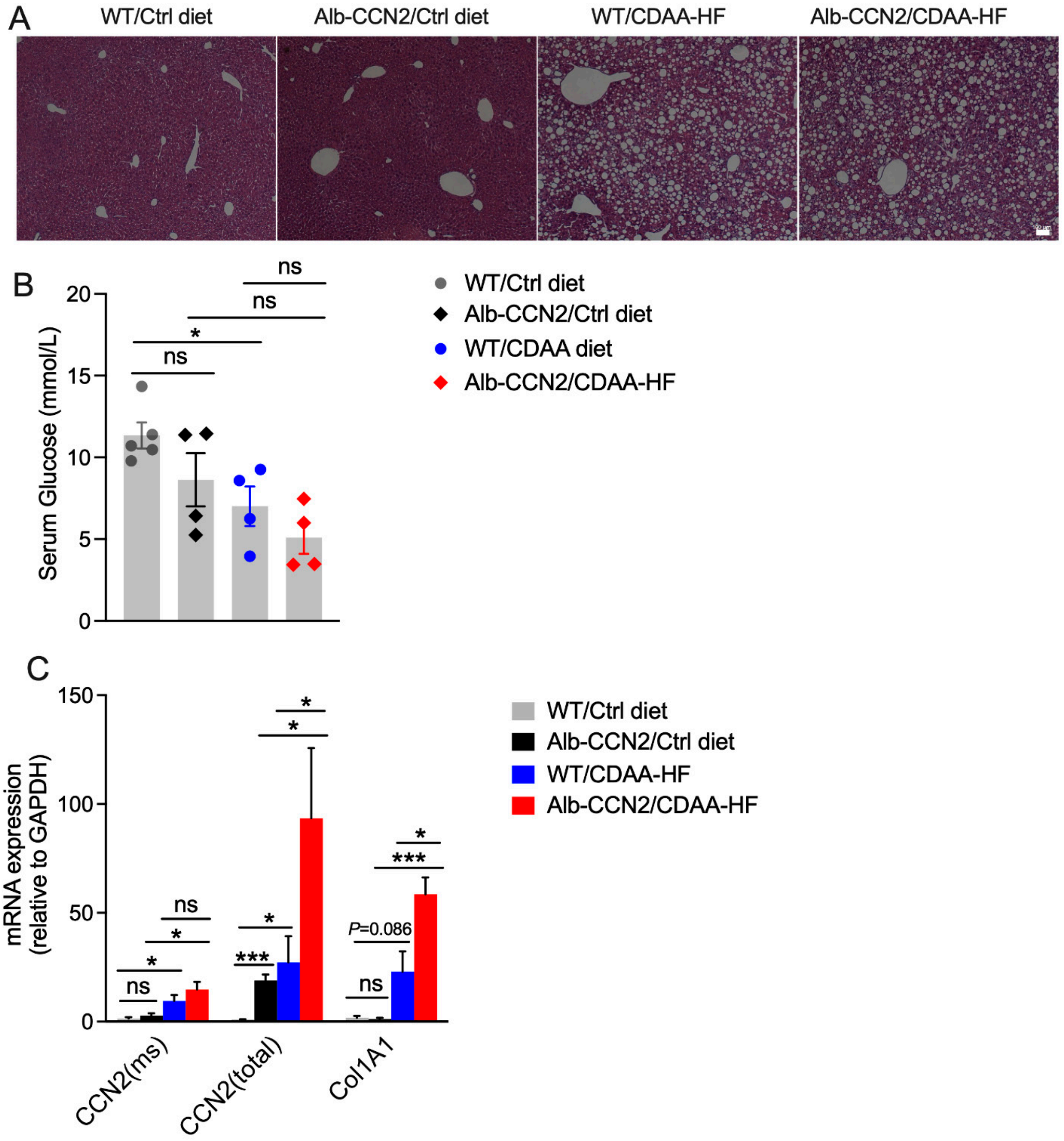

Figure S3. Balb/c WT or Alb-CCN2 mouse liver histology and fibrotic transcript expression. 8-week-old Balb/c WT or Alb-CCN2 were fed with control or CDAA-HF diet for 8 weeks. (**A**) Histology assessed by H& E staining (scale bar = 100μm). (**B**) Serum glucose level measured by using the glucose oxidase-peroxidase (GOD-POD) method. (**C**) Liver tissues were used for total RNA isolation and RT-qPCR detection of mouse CCN2, total CCN2 (human and mouse), and mouse Col1A1 transcript. ns, no significance, * P<0.05, ** P<0.01, *** P<0.005.
